# Suboptimal Patient-Provider Communication About Undetectable = Untransmittable and HIV Transmission Risk in Australia and the US

**DOI:** 10.1007/s10461-025-04783-y

**Published:** 2025-07-03

**Authors:** Sarah K. Calabrese, Martin Holt, David A. Kalwicz, Justino J. Flores, Kaosisochukwu C. Onochie, Benjamin R. Bavinton, Bridget Haire, Anthony K. J. Smith, James MacGibbon, Loren Brener, Timothy R. Broady, John Rule, Bruce Richman, Carla Treloar

**Affiliations:** 1 Department of Psychological and Brain Sciences, George Washington University, 2013 H Street NW, Washington, DC 20006 USA; 2 Department of Prevention and Community Health, George Washington University, Washington, DC USA; 3 Centre for Social Research in Health, University of New South Wales, Sydney, NSW Australia; 4 Kirby Institute, University of New South Wales, Sydney, NSW Australia; 5 National Association of People with HIV Australia, Newtown, NSW Australia; 6 Prevention Access Campaign, New York, NY USA

**Keywords:** Health communication, Sexual and gender minorities, Health personnel, HIV, Sustained virologic response, Undetectable = Untransmittable (U = U), Treatment as prevention (TasP)

## Abstract

The Undetectable = Untransmittable (U = U) campaign aims to raise global awareness that people living with HIV whose viral load is undetectable cannot sexually transmit HIV. Healthcare providers are uniquely positioned to disseminate the U = U message. Our study explored patient-provider communication about U = U and HIV risk from the perspectives of gay, bisexual, and other men living with HIV (MLHIV) and healthcare providers engaged in HIV treatment and prevention service delivery. We conducted 40 semi-structured interviews with key informants recruited through HIV community-based and professional organizations in Australia (*n* = 20) and the US (*n* = 20). Key informants included 20 MLHIV and 20 providers. Data were analyzed thematically. MLHIV were cisgender men aged 29–67 years (M[SD] = 52[13.1]). Providers were cisgender adults aged 30–65 years (M[SD] = 38[9.0]). MLHIV preferred that providers use clear and direct language to explain U = U. When prompted to explain U = U as they would to patients, 8 of 10 Australian and 4 of 10 US providers used language consistent with those preferences. MLHIV, especially US MLHIV, reported that their providers’ explanation of the U = U message was often absent, ambiguous, or inaccurate in practice. Such suboptimal communication aligned with the skepticism about U = U and concerns about patient behavior (e.g., adherence) expressed by several providers in the study. Providers relayed multiple reservations regarding new World Health Organization recommendations about informing patients that low-level viremia (detectable viral load $\leq$ 1000 copies/mL) conferred “almost zero” risk. Many Australian and US providers would benefit from training developed in collaboration with people living with HIV to improve patient-provider communication about U = U and HIV transmission risk.

## Resumen

La campaña Indetectable=Intransmisible (I = I) busca generar conciencia global sobre el hecho de que las personas con VIH cuya carga viral es indetectable no pueden transmitir el VIH sexualmente. Los proveedores del cuidado de la salud están en una posición única para difundir el mensaje de I = I. Nuestro estudio exploró la comunicación entre pacientes y proveedores sobre I = I y el riesgo de VIH desde la perspectivas de hombres gay, bisexuales, y otros hombres que viven con el VIH (HVVIH) y proveedores del cuidado de la salud involucrados en la prestación de servicios de tratamiento y prevención del VIH. Realizamos 40 entrevistas semiestructuradas con informantes clave reclutados a través de organizaciones comunitarias y profesionales del VIH en Australia (n = 20) y los EE.UU. (n = 20). Los informantes clave incluyeron 20 HVVIH y 20 proveedores. Los datos se analizaron temáticamente. Los HVVIH eran hombres cisgénero de entre 29 y 67 años (M[DE] = 52[13,1]). Los proveedores eran adultos cisgénero de 30 a 65 años (M[DE] = 38[9,0]). Los HVVIH prefirieron que los proveedores usaran un lenguaje claro y directo para explicar I = I. Cuando se solicitó a los proveedores que explicaran I = I como lo harían con los pacientes, 8 de 10 proveedores australianos y 4 de 10 proveedores de los estadounidenses usaron un lenguaje consistente con esas preferencias. Los HVVIH, especialmente los de EE.UU., reportaron que las explicaciones de sus proveedores del mensaje I = I a menudo eran ausentes, ambiguas o imprecisas en la práctica. Esta comunicación subóptima se alineó con el escepticismo sobre I = I y las preocupaciones sobre el comportamiento del paciente (p. ej., adherencia) expresadas por varios proveedores en el estudio. Los proveedores transmitieron múltiples reservas con respecto a las nuevas recomendaciones de la Organización Mundial de la Salud sobre informar a los pacientes que la viremia de bajo nivel (carga viral detectable $\leq$ 1000 copies/mL) confería un riesgo “casi cero”. Muchos proveedores australianos y estadounidenses podrían beneficiarse de capacitaciones desarrolladas en colaboración con personas que vive con el VIH para mejorar la comunicación entre pacientes y proveedores sobre I = I y el riesgo de transmisión del VIH.

## Introduction

The Undetectable = Untransmittable (U = U) campaign was launched in 2016 to raise global awareness that people living with HIV (PLHIV) whose viral load is suppressed to an undetectable level cannot sexually transmit HIV [1]. The campaign was preceded by earlier messaging initiatives communicating that HIV treatment offered preventive benefits in the form of reduced HIV transmission risk. In response to mounting empirical evidence that viral suppression to an undetectable level not only reduced sexual transmission risk but eliminated it [2–5], the U = U campaign was pivotal in clarifying and disseminating the message that sexual transmission risk was not low but rather *zero* [1].

The U = U message has gained traction over the years since the campaign first launched, as indicated by growing percentages of people reporting awareness of U = U over time [6, 7]. For many PLHIV, the U = U message has been transformative, reducing self-stigma, alleviating transmission fear, enhancing comfort with having serodifferent sexual partners, reinforcing treatment motivation, and improving retention in care [8–13]. Among HIV-negative people, U = U has been linked to greater openness to having sex with PLHIV, lower HIV stigma, and increased HIV testing motivation [14–18]. Despite the promising implications of U = U messaging for people of both serostatuses, many people remain unaware of U = U. Furthermore, among many people who are aware of U = U, such awareness has not translated to acceptance of the U = U message or understanding that a person with an undetectable viral load cannot sexually transmit HIV [7, 14, 19–21].

Healthcare providers are uniquely positioned to disseminate the U = U message, clarify misconceptions, and bolster message credibility. Previous research has linked provider discussion of U = U to patient health benefits. For example, a 2019–2020 survey of 2389 PLHIV from 25 countries revealed that PLHIV who were informed about U = U by providers had significantly higher odds of HIV treatment adherence; viral suppression; and optimal physical, sexual, mental, and overall health compared with PLHIV who were unaware of U = U [22]. Despite these benefits and their alignment with treatment goals, providers are not consistently communicating about U = U with patients. Early studies have suggested that such communication lapses are sometimes due to providers’ disbelief or lack of understanding regarding U = U despite scientific consensus, discomfort with “zero risk” language, doubts about the relevance of U = U to their patients, perceived liability, or moral qualms about patients changing their behavior (e.g., forgoing condoms) upon learning of U = U [23–26]. Even providers who are aware of U = U and have attempted to communicate about it with patients often lack the language and skills to do so effectively [10, 27]. More research is needed to understand providers’ U = U communication challenges and training needs from the perspectives of both PLHIV and providers.

In this qualitative key informant (KI) interview study, we explored patient-provider communication related to U = U from the perspectives of gay, bisexual, and other MLHIV and healthcare providers engaged in HIV treatment and prevention service delivery in Australia and the US. Australia and the US are two countries in which national clinical guidelines recommend discussing U = U with patients [28–30] but deficits in providers’ U = U knowledge, acceptance, and communication persist [26, 31, 32]. Exploring the perspectives of MLHIV and providers from both countries allowed for cross-cultural comparison.

Our overarching objective was to explore MLHIV’s and providers’ perspectives on provider communication about U = U and HIV risk with patients, including preferences and experiences related to language and other aspects of message delivery. An ancillary objective that emerged partway through the study was to explore providers’ perspectives on if and how a newly published systematic review of scientific evidence, which suggested that the risk of sexually transmitting HIV with low-level HIV viremia was “almost zero” [33], would affect their communication about U = U and HIV risk with patients. The review corresponded to the World Health Organization’s 2023 policy brief that delineated three viral load categories—unsuppressed (> 1000 copies/mL), suppressed (detected but ≤ 1000 copies/mL), and undetectable (not detected by test used)—and advocated new messaging about “almost zero or negligible risk” of sexual transmission for patients in the suppressed category (i.e., patients with low-level viremia) [34].

## Methods

### Participants

A total of 40 KIs (20 MLHIV and 20 providers) were recruited in partnership with community-based and professional organizations in Australia and the US. Specifically, Australian MLHIV (*n* = 10) were recruited by the National Association of People with HIV Australia (NAPWHA), US MLHIV (*n* = 10) were recruited by Prevention Access Campaign (PAC), Australian providers (*n* = 10) were recruited by ASHM (an Australasian organization of HIV, sexual health, and bloodborne virus healthcare professionals), and US providers (*n* = 10) were recruited by the AIDS Education and Training Center (AETC) National Coordinating Resource Center (a program that coordinates national HIV education and training of healthcare professionals).

Eligibility criteria for MLHIV included: English fluency, being 18 years of age or older, living in Australia or the US, being a cisgender or transgender man, having anal sex with a man in the past 12 months, and having been diagnosed as HIV-positive. Eligibility criteria for providers included: English fluency, being 18 years of age or older, currently practicing in a primary care or HIV care setting in Australia or the US, having prescribed antiretroviral therapy for one or more PLHIV in the past six months, and having prescribed pre-exposure prophylaxis (PrEP) for one or more HIV-negative patients in the past 6 months.

### Procedures

All study procedures were approved by the institutional review boards of the University of New South Wales and George Washington University prior to inception. Prospective participants were contacted by email via organizational listservs. The initial email announcement and attached flyer invited them to participate in an interview study about new HIV prevention and treatment options and provided a link to an online screening survey to determine eligibility. Those deemed eligible were contacted about scheduling and provided with additional background information about the study and a verbal consent script for reference during the interview.

The principal investigator (SKC) conducted all interviews by phone or videoconference between February and September of 2023. At the outset of each interview, the verbal consent script was reviewed with the participant, questions about the study were answered, and the participant vocalized their consent. Subsequently, the interviewer asked a series of questions with follow-up prompts using a semi-structured interview guide. For both groups, topics included patient-provider communication about U = U and HIV risk, delivery of new HIV services, potential intersections of U = U and new HIV services with stigma and equity, and provider training needs related to HIV biomedical prevention and stigma. The current analysis focuses on communication about U = U and HIV risk, which included questions about language and content preferences (asked of both MLHIV and providers), past experiences of provider communication about these topics (asked of MLHIV only), and approaches to communicating about these topics with patients (asked of providers only). Additionally, for providers (*n* = 6) interviewed after publication of the literature review reporting almost zero HIV transmission risk with a suppressed (albeit detected) viral load [33] and the corresponding WHO policy brief [34], the interviewer provided a brief overview of the finding and asked if and how the participant would incorporate the new information into their conversations with patients. This topic could only be explored with a subset of six US providers because the review and policy brief were published after all Australian provider interviews and the first four US provider interviews had already been completed.

Following the interviews, participants completed a brief background questionnaire sent via email. The background questionnaire included items related to participant sociodemographic characteristics, HIV treatment, U = U, health and healthcare (MLHIV only), recent sexual and injection drug-related behavior (MLHIV only), and professional background characteristics (providers only). Participants were thanked and compensated with $75 Amazon electronic gift cards in the currency of their country of residence (AUD or USD).

### Analysis

All interviews were audio-recorded and transcribed. Transcripts were cleaned and uploaded into NVivo (Version 14) [35] for management and coding of textual data. Thematic analysis was guided by the Framework Method, a strategy comprising seven specific stages: transcription, data familiarization, coding, development of a working analytic framework, framework application, data charting, and data interpretation [36]. Data charting allowed for structured visualization of the data, facilitating comparisons within and between KI groups (MLHIV and providers) and countries (Australia and the US).

The principal investigator and two research assistants (DAK and JJF) read all transcripts and drafted the analytic framework, which was then refined through an iterative process involving the two research assistants independently coding a transcript using the current version of the framework and then comparing their coding, discussing discrepancies, and revising existing categories and codes or adding new ones as needed. Once the framework was finalized, the two research assistants independently coded a subset of 10 of the 40 transcripts, including transcripts representing both KI groups and both countries, and compared coding to establish intercoder reliability (Κ = 0.93) [37]. Each of the remaining transcripts was coded by a single research assistant.

The principal investigator charted the coded textual data into a matrix and used the matrix to guide data interpretation and select representative quotations. Quotations are presented with the corresponding participant ID and country in brackets. As part of the analysis, the research team judged the relative clarity and accuracy of all 20 providers’ explanations of U = U based on established communication guidelines [30, 38, 39].

### Reflexivity

Reflecting on how the research team’s personal background characteristics and experiences may have shaped a research study, including the questions asked, themes identified, and conclusions drawn, is an essential part of the qualitative research process [40]. The principal investigator of the study was a White, heterosexual, cisgender woman from the US, who conducted all 40 interviews and led data analysis. Research assistants involved in data analysis included a Latino, queer, cisgender man and a White, gay, cisgender man, both from the US. The larger research team was composed of cisgender men and women from Australia and the US and was diverse with respect to sexual orientation, race/ethnicity, and HIV status. Team members were from university settings and community-based and professional organizations, and all had previous experience conducting research related to HIV prevention with MLHIV. The team conceptualized and pursued the research knowing the scientific underpinnings of U = U, recognizing unawareness and skepticism about U = U existed among some providers and community members, and sharing the belief that patients should be accurately informed about U = U by their providers. The interviews were conducted to understand current U = U messaging and to inform future training initiatives to improve providers’ communication skills on the topic and dissemination of the U = U message more broadly. The project was conceptualized and conducted as part of a scholarship through the Fulbright US Scholar Program, an international academic exchange program dedicated to fostering diplomacy across countries in pursuit of shared humanitarian goals.

## Results

### Participant Characteristics

Sample characteristics are presented in Tables 1 and 2, stratified by KI group (MLHIV or provider) and country (Australia or the US). Among the 17 of 20 MLHIV KIs who returned their background questionnaires, ages ranged from 29 to 67 years (M[SD] = 52[13.1]; Mdn[IQR] = 60[23.5]). All identified as gay cisgender men. All reported recently accessing healthcare, currently taking HIV medication, and having an undetectable viral load. All had taken HIV medication in daily pill form. Only one had ever taken it in injectable form (T-20, a twice-daily subcutaneous injection for PLHIV with resistant HIV). Of the 15 MLHIV KIs who responded to questions pertaining to U = U, all reported having heard of U = U and most (67%) reported having ever relied on U = U to prevent HIV sexual transmission.

**Table 1:** Key informant characteristics: men living with HIV

	Australian MLHIV KIs^a^	US MLHIV KIs^a^	All MLHIV KIs combined^a^
	*n* (%)	*n* (%)	*n* (%)
Age (years)			
25-34	3 (33.3)	1 (12.5)	4 (23.5)
35-44	0 (0.0)	1 (12.5)	1 (5.9)
45-54	2 (22.2)	1 (12.5)	3 (17.6)
55-64	2 (22.2)	5 (62.5)	7 (41.2)
65 or older	2 (22.2)	0 (0.0)	2 (11.8)
Gender^b^			
Cisgender man	9 (100.0)	8 (100.0)	17 (100.0)
Sexual orientation^c^			
Gay	9 (100.0)	8 (100.0)	17 (100.0)
Ethnicity			
Aboriginal or Torres Strait Islander^d^	0 (0.0)	–	–
Latino/x^e^	–	0 (0.0)	–
Race^e^			
Black	–	6 (75.0)	–
White	–	2 (25.0)	–
Country of birth^f^			
Australia	4 (44.4)	2 (28.6)	6 (37.5)
US	0 (0.0)	5 (71.4)	5 (31.3)
Other	5 (55.6)	0 (0.0)	5 (31.3)
Education^f^			
Less than bachelor’s degree	4 (44.4)	2 (28.6)	6 (37.5)
Bachelor’s degree or higher	5 (55.6)	5 (71.4)	10 (62.5)
Employment^f^			
Employed	7 (77.8)	6 (85.7)	13 (81.3)
Retired	1 (11.1)	1 (14.3)	2 (12.5)
Unable to work	1 (11.1)	0 (0.0)	1 (6.3)
Health insurance status^f^			
Insured	8 (88.9)	6 (85.7)	14 (87.5)
Uninsured	1 (11.1)	1 (14.3)	2 (12.5)
Most recent healthcare^f^			
Within the past 3 months	8 (88.9)	7 (100.0)	15 (93.8)
4-6 months ago	0 (0.0)	0 (0.0)	0 (0.0)
7-12 months ago	1 (11.1)	0 (0.0)	1 (6.3)
More than 12 months ago	0 (0.0)	0 (0.0)	0 (0.0)
Healthcare settings visited (past year)^f, g^			
Community health center (or healthcare van)	4 (44.4)	1 (14.3)	5 (31.3)
Urgent care or emergency department	4 (44.4)	1 (14.3)	5 (31.3)
Private medical office	7 (77.8)	6 (85.7)	13 (81.3)
Other	3 (33.3)	0 (0.0)	3 (18.8)
HIV treatment experience^f, g^			
Currently taking ART	9 (100.0)	7 (100.0)	16 (100.0)
Current or past use of ART daily pill	9 (100.0)	7 (100.0)	16 (100.0)
Current or past use of ART injectable	1 (11.1)	0 (0.0)	1 (6.3)
Viral load status^f^			
Undetectable	9 (100.0)	7 (100.0)	16 (100.0)
Sex without condoms or PrEP (past 6 months)^f, g^			
1+ partner living with HIV^h^	3 (50.0)	4 (57.1)	7 (53.8)
1+ HIV-negative or status-unknown partner	8 (88.9)	4 (57.1)	12 (75.0)
Shared needles or injection equipment (past 6 months)^f^			
No	9 (100.0)	7 (100.0)	16 (100.0)
Prior U=U awareness/experience^f, g^			
Ever heard of U=U	7 (100.0)	8 (100.0)	15 (100.0)
Ever relied on U=U during sex	6 (75.0)	4 (57.1)	10 (66.7)

*MLHIV KIs* Men living with HIV key informants, *ART* Antiretroviral therapy, *U=U* Undetectable=untransmttable

^a^Data represents 9 of 10 Australian MLHIV KIs, 8 of 10 US MLHIV KIs, and 17 of all 20 MLHIV KIs from the two countries combined due to unreturned background questionnaires; denominators adjusted accordingly

^b^Response options included cisgender man, cisgender woman, transgender man, transgender woman, gender queer or gender nonbinary, and other. Initial screening required identification as a man, cisgender man, or transgender man to be eligible for participation

^c^Response options included lesbian, gay, bisexual, heterosexual, pansexual, asexual, other, and prefer not to say

^d^Presented to Australian MLHIV KIs only

^e^Presented to US MLHIV KIs only

^f^For this variable, *n* < 9 Australian MLHIV KIs and/or *n* < 8 US MLHIV KIs (i.e., *n* = 13-16 total MLHIV KIs) due to missing response(s); denominators adjusted accordingly

^g^Categories not mutually exclusive

^h^For this variable, response patterns for two Australian participants suggest that the absence of a response may have been intended to reflect zero partners, in which case frequency values would be 3 of 8 (37.5%) Australian MLHIV KIs and 7 of 15 (46.7%) total MLHIV KIs

**Table 2:** Key informant characteristics: providers

	Australian provider KIs	US provider KIs	All provider KIs combined
	*n* (%)	*n* (%)	*n* (%)
Age (years)			
25-34	5 (50.0)	3 (30.0)	8 (40.0)
35-44	2 (20.0)	7 (70.0)	9 (45.0)
45-54	1 (10.0)	0 (0.0)	1 (5.0)
55-64	1 (10.0)	0 (0.0)	1 (5.0)
65 or older	1 (10.0)	0 (0.0)	1 (5.0)
Gender^a^			
Cisgender man	7 (70.0)	4 (40.0)	11 (55.0)
Cisgender woman	3 (30.0)	6 (60.0)	9 (45.0)
Sexual orientation^b, c^			
Lesbian	1 (11.1)	0 (0.0)	1 (5.3)
Gay	4 (44.4)	3 (30.0)	7 (36.8)
Bisexual	1 (11.1)	2 (20.0)	3 (15.8)
Heterosexual	3 (33.3)	5 (50.0)	8 (42.1)
Ethnicity			
Aboriginal or Torres Strait Islander^c, d^	0 (0.0)	–	–
Latino/x/a^e^	–	0 (0.0)	–
Race^e^			
Asian	–	3 (30.0)	–
Black	–	2 (20.0)	–
White	–	4 (40.0)	–
Other	–	1 (10.0)	–
Country of birth			
Australia	4 (40.0)	0 (0.0)	4 (20.0)
US	1 (10.0)	6 (60.0)	7 (35.0)
Other	5 (50.0)	4 (40.0)	9 (45.0)
Medical background^f^			
MD, DO, or MBBS	10 (100.0)	5 (50.0)	15 (75.0)
APRN, NP, or RN	0 (0.0)	3 (30.0)	3 (15.0)
PharmD	0 (0.0)	2 (20.0)	2 (10.0)
Type of provider^f^			
General practitioner/primary care provider	6 (60.0)	6 (60.0)	12 (60.0)
HIV specialist^c^	6 (66.7)	10 (100.0)	16 (84.2)
HIV treatment experience^f^			
Ever prescribed ART daily pills	10 (100.0)	10 (100.0)	20 (100.0)
Ever prescribed ART injectables	8 (80.0)	9 (90.0)	17 (85.0)
Prior U=U awareness/experience^f^			
Ever heard of U=U	10 (100.0)	10 (100.0)	20 (100.0)
Ever discussed U=U/viral suppression to prevent sexual transmission with patients^c^	9 (100.0)	10 (100.0)	19 (100.0)

*KIs* Key Informants, *ART* Antiretroviral Therapy, *MD* Doctor of Medicine, *DO* Doctor of Osteopathic Medicine, *MBBS* Bachelor of Medicine, Bacholor of Surgery (MD equivalent), *APRN* Advanced Practice Registered Nurse, *NP* Nurse Practitioner, *RN* Registered Nurse, *PharmD* Doctor of Pharmacy, *U=U* Undetectable=Untransmttable

^a^Response options included cisgender man, cisgender woman, transgender man, transgender woman, gender queer or gender nonbinary, and other

^b^Response options included lesbian, gay, bisexual, heterosexual pansexual, asexual, other, and prefer not to say

^c^For this variable, *n* = 9 Australian providers and *n* = 19 total providers due to ambiguous or missing response; denominators adjusted accordingly

^d^Presented to Australian providers only

^e^Presented to US providers only

^f^Categories not mutually exclusive

Provider KIs, all of whom returned their background questionnaires, ranged in age from 30 to 65 years (M[SD] = 38[9.0]; Mdn[IQR] = 35.5[7.5]). All identified as cisgender. The provider sample was composed of 35% gay men, 25% heterosexual women, 15% heterosexual men, 10% bisexual women, and one of each of the following: lesbian woman (5%), bisexual man (5%), and woman of unspecified sexual orientation (5%). Most provider KIs reported being trained as medical doctors (75%). All had prescribed HIV medication in pill form, and most (85%) had prescribed it in one or more injectable forms. All reported having heard of U = U and having discussed U = U or viral suppression to prevent HIV sexual transmission with one or more patients; the number of patients with whom they discussed U = U or viral suppression ranged from 11 to over 1000 (M[SD] = 321[291.2]; Mdn[IQR] = 250[300]).

### Main Themes Related to Patient-Provider Communication about U = U and HIV Risk

**Overview**: MLHIV KIs from both countries indicated that providers should—but do not consistently—communicate the U = U message using clear and direct language. They recommended that messaging be tailored to the health literacy of individual patients, supported by supplementary resources, and delivered to both PLHIV and HIV-negative/status unknown participants. When provider KIs were prompted to explain U = U/HIV risk as they would to patients, the majority of Australian providers and a minority of US providers used clear, accurate language. Those who did not do so expressed skepticism about U = U and concerns about patient behavior.

**MLHIV**: MLHIV KIs from both countries expressed a preference for providers to discuss U = U using language that is clear (i.e., easy for patients to understand) and direct, definitively communicating sexual transmission risk to be zero. As explained by one participant:

It’s very important that the language used is really straightforward, everyday talk. It’s not riddled with science jargon … the way it is described should be, “Look, here’s the reality. If we get you on medicine … and we get you to this healthy, undetectable level, which means the virus is so, so low in your body and controlled, you cannot transmit the virus, you just can’t.” [MLHIV KI 11, US]

Another participant highlighted the importance of plain language for patients born overseas:

A lot of the people that are still getting HIV in Australia are those ones who have migrant experiences and English is not their first language. And you bring up a term like “virtually [no risk].” We are like, “What do you mean ‘virtually,’ like … ?” So, simple and direct language definitely helps. And then, you know, some doctors like using the big words like “virtual transmission.” It’s not such a big word when you work in this environment, but for your average community person on the street, like, you know, they are not always that clear. [MLHIV KI 4, AUS]

Over half of the MLHIV KIs suggested that providers could strengthen their messaging about U = U with the use of visual aids, take-home educational materials, or other supplementary resources.

Despite the language preferences expressed, MLHIV KIs reported that the U = U message was absent, ambiguous, or inaccurate in most conversations they had previously had with providers, including conversations that had transpired in the years since scientific studies had conclusively demonstrated U = U (see Table 3 for examples). The failed messaging was common among men in both countries, but it was especially prevalent in the US, where only one participant [MLHIV KI 20, US] reported that their provider had clearly delivered the U = U message to him. Several participants responded with anger and frustration to perceived messaging failures. One participant recounted confronting his provider upon learning about U = U from an HIV activist at a time when scientific evidence for U = U had already been established, expressing the belief that the provider had a professional responsibility to relay these scientific findings even if health authorities had not yet formally endorsed U = U:

I spoke to my doctor. I asked why no one had talked about this, and it was because, you know, it hadn’t sort of been officially said by the CDC or the World Health Organization. So, my doctor acknowledged that he knew about it but chose not to talk to me about it … I was pretty pissed off. I was annoyed. I was annoyed … It would have been very helpful for me … to be told because it gives someone hope, and it changes—you know, it’s one thing to be told by doctors that HIV is not a death sentence anymore. Um, it’s another thing … to think that, you know, sort of part of me has been cut off in a way that really, that anytime I’m gonna have sex with somebody it has to be with a condom, and, even then, there are still risks when now we know the reality is that, um, as long as I’m undetectable, there’s zero risk. It’s a huge mental shift. [MLHIV KI 11, US]

**Table 3:** Experiences of suboptimal provider messaging about U=U reported by men living with HIV

Nature of U=U messaging failure	Example (response quoted verbatim)
Absence	“I don’t discuss [HIV transmission risk] with my doctor … I’ve got friends … I get information from them, but I don’t actively seek information or discuss it with my healthcare provider. I’ve been seeing the same chap for many years now. I don’t think we’ve ever had a discussion about … transmission.” [MLHIV KI 7, AUS]
	“I didn’t know about Undetectable=Untransmittable until about four years ago when I chose to publicly disclose my status, and then someone reached out to me on social media. And that was the first time I’d heard of U=U, but, up until that point, I’d been living with HIV for ten years at that point. So, it wasn’t from a doctor that I heard it from. It was from an HIV activist.” [MLHIV KI 11, US]
Ambiguity	“They have said that it’s extremely unlikely … They are sort of accompanying information that sort of says that undetectable is untransmittable … They do stress it is extremely unlikely and that the information that goes out is that it doesn’t happen … ” [MLHIV KI 3, AUS]
	“Doctors would be always on the safe side, there’s always like it’s not full-on, like you haven’t encountered any doctors in Australia that would say U=U, like admittedly would say that because I feel that there’s still a lot of reservations and just to I guess … for example, the last conversation I had with my doctor about U=U I think two to three years ago, it was still very vague. I, we kind of discussed like transmission risk and how my undetectable status is and how I … the doctor acknowledging that, you know, ‘You can’t transmit the virus anymore with an undetectable status, however … ’ there’s the ‘but,’ like, ‘but still use, you know, precautionary methods like condoms or like ask your partners, sexual partners, to use PrEP or PEP.’” [MLHIV KI 5, AUS]
Inaccuracy	“I’ve been undetectable pretty much since the beginning [when diagnosed 18 years ago] … We’ve talked about it, we talked about being undetectable, although undetectable, the chances are very slim that you can transmit.” [MLHIV KI 15, US]
	“‘The risks are much lower from you transmitting [to your monogamous sexual partner who is also an MLHIV] or him transmitting you because you’re both undetectable’ … I’ve never gotten a total zero … I would love to feel zero risk, and if a professional said it to me, I probably would be more inclined to say. But since that’s never happened, I’m kinda, I think I’m on the fence with it.” [MLHIV KI 16, US]

*MLHIV KI* Men living with HIV key informant, *U*=*U* Undetectable=Untransmittable, *PrEP* Pre-exposure prophylaxis, *PEP* Post-exposure prophylaxis

When asked about the extent to which providers had talked with him about HIV transmission risk, another US MLHIV KI similarly referred to his providers’ neglect to convey the U = U message as being a missed opportunity for psychological benefit:

I could be wrong, but I don’t believe that has been discussed. So, I’m fully aware of U = U, but I got that information from elsewhere … I’m not sure that there’s a realization of what a big deal it is for the person with HIV from the provider side. Like … maybe they don’t realize that it’s such a huge mental relief to people with HIV. [MLHIV KI 13, US]

Several MLHIV KIs also raised the relevance of provider assumptions about a patient’s pre-existing knowledge to U = U message delivery. They expressed concern about providers neglecting to tailor the message to patients with limited pre-existing knowledge about HIV, thereby failing to communicate the information effectively:

They should know the audience as well because it can be quite mixed. Because I might be coming in on the baseline and someone is on a much higher level or lower level. So, it should be able to capture the lot, not assume things. Don’t take it for granted. [MLHIV KI 8, AUS]

On the other end of the knowledge spectrum, a few MLHIV KIs believed that their provider had never raised U = U with them because their provider assumed they had preexisting knowledge (given their involvement in HIV activism/education). However, such an assumption was not necessarily considered an acceptable justification.

MLHIV KIs perceived there to be a number of benefits associated with providers informing not only PLHIV about U = U, but also HIV-negative and status-unknown patients. Benefits included decreasing stigma, reducing anxiety about HIV transmission, reducing fear of HIV testing, equipping people to cope with a future HIV diagnosis, increasing openness to serodifferent partnering, facilitating HIV-related conversations between serodifferent partners, and enabling U = U message dissemination through patients’ social networks. Several MLHIV KIs highlighted the importance of healthcare providers being a source of the U = U message given their health expertise: “Healthcare providers … are looked at as authority figures … For the person who didn’t know about [U = U], there was some slight skepticism … there was like side-eye … they want more assurance than just coming from me” [MLHIV KI 18, US]. Although many MLHIV KIs favored providers educating all patients about U = U, a few favored providers selectively educating HIV-negative/status-unknown patients about U = U based on patient inquiry or perceived patient risk.

Finally, a couple of MLHIV KIs expressed concerns about the potential inadvertent harm that could come from a provider communicating about U = U without proper nuance or sensitivity. As explained by one participant:

[The U = U message] created a minority within the community of those people who can’t achieve a total undetectable viral load when they think that an undetectable viral load should be 0. So, that caused quite a lot of anxiety for people who weren’t achieving what they thought was an undetectable viral load and were stressing out. One particular example of that was that young man … he’d been sitting on a viral load of 176, and he was absolutely distraught about the inability of being able to achieve an undetectable viral load, and it wasn’t until I sat down with him and went through the very complex science with him and explained that he was still undetectable, even though he was registering on the test. Getting that message to him was really difficult because he was going, “But it’s not saying 0” … So, it does confuse and it does upset people. [MLHIV KI 9, AUS]

**Providers**: Consistent with MLHIV KI’s expressed preferences, 8 of 10 Australian and 4 of 10 US provider KIs used language that the research team considered to be relatively clear and accurate when prompted to recount the way that they typically explained U = U or the implications of undetectability for HIV sexual transmission risk to patients. Table 4 presents the specific responses that providers gave.

**Table 4:** Provider explanations of U=U to patients

Country	Participant	Explanation^a^ (response quoted verbatim)
Australia	Provider KI 1	“Being undetectable … meaning that you cannot pass on your HIV to anybody.”
	Provider KI 2	“If your viral load is undetectable, then you can’t sexually transmit the virus, so you can have sex with anyone you want and they won’t get HIV … as long as you stay on your treatment and you remain undetectable.”
	Provider KI 3	“Shitloads of virus, really easy to get infected; hardly any virus, really hard to get infected; no virus we can find, you can’t [get infected] … You can’t actually have immaculate infections … You know, you can’t have spontaneous transmission from nothing.”
	**Provider KI 4**	**“Once people have been on treatment for sort of 6 months and they are undetectable, then we know that the risk of partner transmission as long as they are consistent with their treatment and coming in for their regular check-ups is really negligible, so I do let people know that … once we get to that sort of undetectable six-month mark, we know with U=U that that their risk is going to be really none. I think the message to patients is ‘There is no risk, that if you are undetectable, you are taking your medicines, you are coming in for your blood tests, there is no risk.’”**
	Provider KI 5	“I say, ‘There’s no risk,’ and I say that to the patients … So, if their partner is sitting there, I tell them, ‘As long as [the patient is] undetectable, there’s no risk, you’re not going to [get HIV].’”
	Provider KI 6	“If there’s no virus circulating, then there’s no way of catching it, because it’s not being transmitted.”
	Provider KI 7	“If you’re undetectable in the bloodstream, that it can’t spill over into things like semen and like other bodily fluids and therefore you can’t pass it on to somebody else … It’s not possible based on the research, we know, it’s not possible to pass it on, you can’t, won’t be able to pass it on through sex.”
	**Provider KI 8**	**“[I] would be confident in saying if they have undetectable viral load, that they’re consistent in that, that they had, you know, taken their antiretrovirals regularly and on a consistent basis and their blood tests showed, you know, a stable CD4 count and an undetectable viral load consistently, then I’d be very confident that they would be at no risk, well, little-to-no risk of transmitting to others … It’s not an exact science. You can’t know for total certainty, but we can be, I can be confident in telling the patient or telling you in front of me that your risks of transmitting to others [is] going to be negligible.”**
	Provider KI 9	“If you are compliant to medications; you have not missed anything; you have not stored it in a very hot temperature, in a car; and your medications work just fine because there’s no heat problems and all of that and you always have undetectable viral load that’s 200 and below copies, then you cannot transmit that risk to anyone at all.”
	Provider KI 10	“When you’re undetectable for three months, you can’t transmit an HIV infection through sexual contact, despite your best efforts … once you’re on medication, you know, we’re going to get you undetectable, which means … you’re not going to be putting any of your sexual partners in any danger at all.”
US	**Provider KI 11**	**“It’s like 99%. You know, it decreases your risk.”**
	**Provider KI 12**	**“We talk about undetectable meaning having viral load of less than 200 copies in their bloodstream … once they’re under that number, that means that their risk of transmitting HIV to their partners is virtually 0% … I always educate them, saying that, ‘U=U, you know, we can, we can really trust that you’re not at risk.’”**
	**Provider KI 13**	**“If you’re undetectable, like the viral load is less than 20 copies per ml in your blood, then the odds of you transmitting it sexually or in any other way to your partner if you’re having unprotected sex are like close to zero … the chances are extremely low or very low that you transmit it to your partner since you’re undetectable.”**
	**Provider KI 14**	**“I use the exact words, I use ‘undetectable equals untransmittable’ … ‘So, once you reach undetectable, so recently passing the virus through your sexual partners, even that use of condoms, it’s unlikely almost based on the study … nothing is 100% in science … as long as you are taking the pill every day, it keeps you undetectable and you’re not able to pass the virus.’” [see longer excerpt in main text]**
	Provider KI 15	“We’ve done lots and lots of studies … what we found is that among people who have HIV, who are taking their HIV medication consistently, and when we do a blood test to see can we measure how much HIV is in the blood, we can’t detect it anymore. They still have HIV, but we can’t detect the virus, those folks are not able to transmit HIV to their sexual partners.”
	**Provider KI 16**	**“‘If you are taking your meds and you are undetectable … you are essentially not at risk.’ Putting anyone else at risk of contracting HIV … we say it’s like a 99%. Right. So as I said, yeah, essentially, no risk of transmitting it if they’re being on ART. I don’t want to say 'no' completely. No, you know, it’s 98 to 99%.”**
	**Provider KI 17**	**“‘If your viral load is undetectable for at least six months, the risk of transmitting HIV is close to zero. Basically zero,’ I say, ‘basically zero.’”**
	Provider KI 18	“As long as they’re under 200 copies … then HIV is not able to be transmitted to others in terms of sexual contact.”
	Provider KI 19	“The data that supports this, that it’s a viral load under 200. In that, the more that if you take your medications, and you get there, you can’t transmit to any partners … you cannot transmit the virus um if your viral load is under 200.”
	Provider KI 20	“There’s no risk in terms of sexual transmission, if they’re taking their medications, and they’re undetectable … I say ‘zero risk’ or ‘absolutely no risk.’”

*KI* Key informant, *U*=*U* Undetectable=Untransmittable, *ART* Antiretroviral therapy

^a^Responses given when prompted to explain U=U/HIV risk as they would to patients. Relatively unclear or inaccurate explanations, including explanations that contain both clear/accurate and unclear/inaccurate content, are presented in bold font

Provider KIs who delivered the U = U message clearly and directly, using language such as “cannot pass on,” “no risk,” and “not able to transmit,” often went on to explain associated parameters, like the necessity of sustained medication adherence. For example, one provider stated:

I frame it around, especially if it’s somebody who’s sexually active, feeling confident in the types of sex that they want to have. Recognizing, of course, the limits to do U = U, in that like you have to take your tablets and it does need to be a certain length of time after diagnosis. [Provider KI 7, AUS]

Explanations of associated parameters such as these provided patients with important details without undermining the U = U message.

By contrast, other provider KIs used tentative language that implied sexual HIV transmission risk still existed, describing the risk as “negligible,” “99%,” or “very low.” These providers had different rationales for implying risk. Some, including those familiar with the scientific evidence underlying U = U, did not fully believe in the concept of U = U and objected to the use of terminology that was absolute or conveyed infallibility, making assertions such as “You can’t know for total certainty” [Provider KI 8, AUS].

Another group of provider KIs who communicated to patients that sexual transmission risk existed despite sustained viral undetectability seemed to waver in their beliefs or hold incongruent beliefs about U = U: “Nothing’s ever 100% in healthcare, but we do know from the studies that, you know, if they are suppressed, and they stay on therapy, that they can’t pass it on sexually” [Provider KI 11, US]. Dissonant beliefs such as these seemed to translate to convoluted messaging about U = U to their patients:

I use the exact words, I use “undetectable equals untransmittable … So, once you reach undetectable, so recently passing the virus through your sexual partners, even that use of condoms, it’s unlikely almost based on the study.” … I always tell patients, you know, “Science is a science, right? But nothing is a 100% in science.” So, I don’t give, I don’t know, I- yeah, because I’m just using the words from the, from the resea- I don’t, I don’t want to add that I want to give them 100% assurance that everything is 100% because really nothing is 100% in science … But, yeah, I tell them, “It gives you the highest level of protection. You know, as long as you are taking the pill every day, it keeps you undetectable and you’re not able to pass the virus.” [Provider KI 14, US]

Other provider KIs who conveyed sexual transmission risk existed at undetectable viral load levels believed in the concept of U = U but avoided the use of “zero risk” language because of concerns about patient behavior. One behavioral concern was nonadherence, which could change a patient’s viral load and consequent risk of transmission. As one provider explained:

[Viral load] can change if you’re not 100% adherent, right? So even if you miss two days of your medicine, so you say, “Yeah, I’m on my meds, I’m always taking meds” … Some people, you know, they- they don’t take it at the, you know, every 24 hours for a couple of days, or, you know, just changing the way that they- they take the medicine themselves that could potentially change the risk … I think that can change really quickly. [Provider KI 16, US]

Another provider who believed in the U = U concept chose to inform patients that the risk was “close to zero” or “extremely low” due to concerns that patients might change their behavior:

… just for like harm reduction purposes, so they don’t just, like, go out and be like, okay, I’m undetectable, I can do whatever I want, and I can, you know, like, share my needles and, like, have an orgy with people. [Provider KI 13, US]

Notably, these examples of “harms” that the provider aimed to reduce—needle sharing and participation in orgies—were behaviors that were not directly relevant to U = U (which is specific to sexual transmission) or not inherently unsafe, respectively.

Although much of the discussion about U = U message delivery with provider KIs focused on the level of transmission risk conveyed, one of the US providers raised a concern that was similar to the concern expressed by MLHIV KIs regarding the potential inadvertent harm that could arise from U = U messaging if not delivered sensitively. She also conveyed the ongoing effort required to attain and maintain an undetectable viral load status, and how it could be exacerbated by social disadvantage:

In more recent years, I’ve been doing more work with some of the Black queer community. And there’s been a lot of discussion about how privileged access to HIV medications is, and there’s sort of a hierarchy, if you will, sort of being established between people who are undetectable with people who are not, and the people who are not are thought to be, you know, irresponsible, and, you know, sort of infectious and like transmitting infection into the community. And so now, I try to still offer that- that information [about U = U], because I think it’s really important and helps to fight against stigma, but I also make a point of emphasizing that like getting undetectable, is certainly a process … I’ve tried to be more mindful of the way I talk about it. [Provider KI 15, US]

### Preliminary Insights Related To Incorporating “Almost Zero” Risk Messaging into the U = U Conversation

An ancillary objective that emerged partway through the study was to explore provider KIs’ perspectives on incorporating newly published evidence suggesting that HIV sexual transmission was rare but possible for PLHIV who had a suppressed but detectable viral load (200-1000 copies/mL) [33] into their conversations about U = U and sexual risk with patients. Given the timing of the publication, only six US providers were asked to share their views. (All other provider KI interviews had already been completed.) Of the six, three reported having heard of the finding previously—one from the published article, one via a conference presentation, and one from colleagues. Two reported that the finding was new to them, and one did not specify their prior awareness of the finding.

Provider KIs universally expressed interest about the finding, but they varied in the levels of concern and enthusiasm that they conveyed with respect to sharing this information with their patients. A primary concern was the potential for patient confusion. As one provider explained:

I don’t even think we have the language and, like, strategy for communicating, even, like, more clearcut U = U to people and so to add any, like, “Oh, but if your level is 600 to 1000 … ” What are you talking about? Like, who’s going to understand that? [Provider KI 15, US]

The provider indicated that they had not informed any of their patients about the new information and were unlikely to start doing so given the resultant patient confusion that they anticipated. Another provider concurred regarding the complexity of messaging on the topic of HIV sexual transmission risk relative to viral load, stating:

We have been so careful with language … I don’t want to be the, you know, the public relations person responsible for that particular program, because how do you go from undetectable[= untransmittable] to now … taking it back a little bit and saying, “Well, not necessarily undetectable. But even if your viral load is only 400, which isn’t undetectable … that’s also still fine.” I think it’s just really, it’s challenging. [Provider KI 20, US]

An additional concern that provider KIs expressed related to the instability of viral loads in the virally suppressed but detectable range (200–1000 copies/mL), and the potential for patients who receive a test result in this range to incorrectly assume that they remained in that range and retained minimal risk of sexual transmission. One provider explained:

I think what we see is that there’s a big swing within those patients that are above 200, that sometimes they’re starting to be above 200. But really, they’re off their meds completely. So, they could be going up at that time … I worry about that, like, consistency of the patients sitting within that range … What about a month from now, or two months from now … they’re completely off their medications, and they’re at a million copies. [Provider KI 18, US]

The same provider went on to raise concern about viral resistance and transmission thereof, describing the following hypothetical scenario:

If they’re intermittently adherent, and they start to get resistance, they could develop more and more resistance, because they’re not taking that medication consistently … and have a chance of passing on that resistant virus eventually. [Provider KI 18, US]

Provider KIs also indicated that they perceived the new information to be of limited relevance to most of the PLHIV whom they treated. Explaining the transient nature of a viral load in the virally suppressed but detectable range, one provider stated:

Usually there’s a problem if their viral load is between 200 and 1000 … we’re not going to consistently let someone stay at a viral level like 600, either. Yeah, either they’re not taking [medication] or we’re switching their meds. It’s not just like a state people are staying. [Provider KI 16, US]

The provider noted that the relevance of the new information was not just time-limited for a given patient, but also—as suggested by multiple other providers—not directly relevant to most patients who were adherent to their treatment regimen: “It’s very unlikely we would see a blip like that, like, for no reason … like, a normally adherent person to a good medication regimen is not gonna, that’s not going to be a common occurrence” [Provider KI 16, US].

Despite concerns about, patient confusion, viral load fluctuation, viral resistance, and relevance, most provider KIs expressed openness to communicating the finding of “almost zero” risk in select cases, such as with patients in or around the 200-1000 copies/mL range [Provider KIs 19 and 20, US] or with patients who inquired [Provider KIs 16 and 17, US]. One provider [Provider KI 20, US] spoke of the message also being valuable to communicate to PLHIV more broadly:

But I do think it’s important because we do have patients who are living with HIV who may have you know, blips or sort of low-level viremia, from time to time, you know, life happens if they are suffering from depression, and, you know, just couldn’t take their meds for a week or something. And they had, you know, a little bit of a rebound. I think it is empowering and freeing, and also helps people’s kind of conscience and their own sort of guilt that they put themselves through to know it’s still okay, that they’re not going to transmit HIV to someone if it was during that period of time that they were struggling with adherence. So, I think it is a really important message for us to put out there for people living with HIV. [Provider KI 20, US]

The same provider noted the potential reassurance this information could provide to HIV-negative partners of PLHIV with suppressed but detectable viral loads.

## Discussion

Current professional guidelines in Australia and the US call on providers to discuss U = U with all of their patients living with HIV [28–30] and have done so since at least 2020 [30, 41]. In our study, MLHIV KIs from both countries supported this directive but commonly described the messaging related to U = U that they had received from their providers, if existent, to be ambiguous or inaccurate. They highlighted the importance of providers using clear and direct language and tailoring the message to a patient’s level of health literacy. The majority of Australian and a minority of US provider KIs used language that was consistent with the reported preferences of MLHIV KIs in the study when prompted to explain U = U/HIV risk as they would to patients. The others opted to use more tentative language that implied sexual transmission risk persisted at sustained, undetectable viral load levels. Ambiguous or inaccurate messaging about U = U was more commonly expressed by US provider KIs and reported in the accounts of US MLHIV KIs compared with their Australian counterparts in our study.

Providers’ use of ambiguous and inaccurate language when communicating about HIV risk has been reported in other provider samples [27, 31]. Similar to provider KIs in our study, Australian HIV service providers interviewed in 2019–2020 varied in their description of sexual transmission risk associated with PLHIV whose viral load is undetectable, with some using language such as “extraordinarily low” or “negligible” [31]. For several provider KIs in our study, skepticism and ambivalence about U = U underpinned their miscommunication of the message, corroborating recent survey data documenting variable levels of agreement with U = U among providers in both countries [26, 32]. Overcoming provider skepticism about U = U is an essential precursor to effective patient-provider communication about U = U [26].

According to professional standards in both Australia and the US, healthcare providers have an ethical imperative to ensure that their medical knowledge is up to date and that they obtain informed consent from patients prior to treatment [42–44], suggesting that providers should have accurate knowledge about U = U and relay this treatment benefit to the PLHIV whom they treat. Both countries have also produced clinical guidelines and other professional resources to encourage providers to discuss U = U with their patients; many of these resources use clear and direct language to explain the concept of U = U [1, 30, 38]. For example, Australian professional guidelines published by ASHM state that maintaining an undetectable viral load (< 200 copies/mL) “eliminates” the risk of sexual HIV transmission and recommend that providers inform patients that “People who keep their HIV viral load at an undetectable level by consistently taking HIV medications will not pass HIV to others through sex” [30]. However, other currently available resources may allow confusion or skepticism to persist among providers [45]. The current HIV treatment guidelines published by the US Department of Health and Human Services, which explicitly name U = U and endorse patient education about U = U, technically recommend that all patients living with HIV be informed that maintaining an undetectable viral load (< 200 copies/mL) “prevents” sexual HIV transmission [29]. “Prevents” is a term that may suggest risk reduction rather than complete elimination, just as contraception “prevents” pregnancy and sunscreen “prevents” skin cancer. Based on the significance of word choice highlighted in our study, we recommend that these and other resources be carefully reviewed and revised to eradicate any doubts among providers.

Although U = U skepticism accounted for suboptimal U = U messaging among some provider KIs in our study, others expressed belief in U = U but intentionally distorted the U = U message because of their concerns about patient behavior. Specifically, they expressed concern about their patients’ ability to maintain an undetectable viral load (e.g., due to suboptimal adherence) or about their patients engaging in behavior that they deemed risky or disapproved of in response to learning that sexual transmission risk was zero. Doubt in patients’ ability to understand, manage, and apply the concept of U = U; associated concerns that patients will attempt to rely on U = U for protection when their viral load is not actually undetectable, thereby leading to incidental transmissions for which providers will be held responsible; and the potential for patient behavior change (e.g., engaging in more sex or less condom use) have all been reported previously as barriers to U = U communication [11, 24, 25, 32]. Importantly, obfuscating the U = U message for any of these reasons is medically unjustified. Moreover, doing so deliberately because of anticipated patient behavior is paternalistic and dishonest, akin to withholding PrEP, contraception, or other biomedical interventions for this reason [46]. Misinformation about U = U and HIV risk could compromise PLHIV’s quality of life and, if they become aware that information was intentionally misrepresented or withheld, erode their trust in the healthcare system.

Providers’ reluctance to assert that there is *zero risk* of PLHIV with an undetectable viral load sexually transmitting HIV has been reported elsewhere [23] and deserves further consideration. Providers have sometimes attributed this reservation to the uncertainty inherent in science and the notion that “nothing is 100%” [Provider KI 14, US]. However, for decades, providers have routinely reassured patients that other behaviors, such as hugging or sharing eating utensils, carry zero risk. Ironically, these reassurances have been offered based on far less robust scientific evidence than the solid evidence base that has amassed in support of U = U. It is possible providers’ long-standing belief that condomless sex always carries some risk of transmission has become so ingrained that it is difficult for providers to recalibrate. It is also possible that providers’ hesitancy to relay the “zero risk” message is driven by the same deep-seated conservative sexual values and HIV stigma that likely drive distortion of the U = U message for fear of patient behavior change.

One provider KI and a couple of MLHIV KIs highlighted the potential inadvertent harm that could arise from messaging related to U = U, including unnecessary distress for PLHIV with undetectable viral loads who misunderstand the “undetectable” threshold (e.g., as zero copies/mL) as well as stigma experienced by PLHIV whose viral load is above the threshold (“detectable”). A recent study with US gay, bisexual, and other men who have sex with men [19] documented confusion surrounding the notion of undetectability, including the misconception that “undetectable” meant zero copies/mL, 50 copies/mL, or another value besides the 200 copies/mL threshold that has been established based on key studies [2–5]. The confusion and unnecessary anxiety experienced by patients whose viral load levels are below 200 copies/mL but erroneously believe their viral loads to be above the threshold of transmissibility has led some scholars to call for reforming the current reporting system for viral load test results by adding interpretations alongside numerical results (e.g., “no risk of sexual transmission”), replacing numerical values with qualitative labels (e.g., “undetectable”), or ensuring there is preemptive counseling about the 200 copies/mL threshold [47].

The risk of the U = U message causing inadvertent distress and consequent need for sensitivity in its delivery may be amplified for patients experiencing social marginalization that undermines access or adherence to antiretroviral therapy. The provider KI who linked inequity in access to antiretroviral therapy (and thus viral load undetectability) to her Black queer clientele spoke to the further devaluation and villainization that PLHIV have encountered because of their detectable viral load status. By promoting and idealizing an undetectable viral load status, the U = U message can unintentionally perpetuate stereotypes of people with a detectable viral load status as being irresponsible and infectious [9, 13, 48]. This risk of patient harm as well as a myriad of social and structural barriers to antiretroviral therapy access and adherence [49, 50] introduce added layers of complexity for providers communicating about U = U. Consultation with PLHIV and other community members may help providers to navigate this complexity and to tailor the message appropriately for PLHIV whose viral load is unsuppressed, suppressed (detected but ≤ 1000 copies/mL), or undetectable, as well as for HIV-negative/status-unknown individuals.

As HIV science evolves, communicating new information about HIV transmission risk relative to different behaviors (sex, injection drug use, breastfeeding) and viral load thresholds will pose ongoing challenges for providers. In our study, we explored provider perspectives on the 2023 report that transmission risk was rare but possible at suppressed viral load levels (200–1000 copies/mL) and associated recommendations around communicating “almost zero or negligible” risk. The subset of US provider KIs with whom we spoke about this topic cited multiple concerns about such communication, including the potential for patient confusion, viral load fluctuations, and viral resistance, and questioned the relevance of this information to most of their patients living with HIV. Despite these reservations, most of the provider KIs expressed openness to communicating the finding in select cases in which it was deemed directly applicable or in response to specific inquiries.

Many of the insights about U = U patient-provider communication that were shared by MLHIV and provider KIs in our study relate to the principles described in the World Health Organization’s Strategic Communications Framework [39]. The framework suggests that communication will be most effective when the information is accessible, credible/trusted, understandable, relevant, actionable, and timely [39]. Providers routinely and broadly delivering the U = U message in their clinical practice can help to maximize its accessibility to potential beneficiaries, including those living with or without HIV, and ensure that it is received from a credible source. Using simple and straightforward language when explaining U = U, potentially supported by visual aids or other supplementary resources [51, 52], can improve the understandability of the message, particularly if the message is not being delivered in the patient’s primary language. Tailoring message delivery according to each patient’s HIV status, health literacy, and experience can enhance relevance. Discussing the implications of U = U for individuals’ sexual health decision-making can make the information immediately actionable. Finally, delivering the message as early as possible (e.g., during an initial patient visit) can enable patients to receive the message and associated benefits in a timely manner and avoid subsequent perceptions of information/benefits having been intentionally withheld.

There was a notable discrepancy in provider KIs’ reported communication about U = U between the two countries, with most (8 of 10) Australian providers conveying the U = U message in an accurate way when prompted compared with half as many US providers. Likewise, only one US MLHIV KI recounted clear communication of the U = U message from a provider. This pattern is consistent with the disparate rates of retention in care and viral suppression in the two countries when considered in the context of early evidence for U = U messaging in healthcare being associated with greater retention in care and viral suppression [10]. However, the extent to which the discrepancy in communication between Australian and US provider KIs that we observed in our study is related to the national discrepancies in clinical outcomes cannot be determined from this study. The significant gap in healthcare access and affordability between the two countries as well as cultural differences likely play a prominent role. Nonetheless, the cross-cultural design of the study highlights communication strengths and areas for improvement in two countries on opposite sides of the world, with insights from each potentially instructive to the other.

### Study Limitations

Our study is not intended to generalize to the larger populations of providers and MLHIV in the US and Australia and should be interpreted accordingly. For example, providers in our study were selected as KIs based on their professional affiliation and experience providing HIV treatment. U = U knowledge and communication are likely to be even more variable in broader populations of providers that include providers with less treatment experience. Provider knowledge and communication are also likely to vary by geographic location and medical setting. Although this information was not systematically collected from all study participants, many providers—including most US providers—reported practicing in urban settings.

Our samples of MLHIV and provider KIs were not recruited as patient-provider dyads or from the same health centers. Thus, they were not reflecting on experiences with one another in particular, and discrepancies in their perspectives should be interpreted with this in mind. For example, we cannot infer that the clarity of most Australian provider KIs’ delivery of the U = U message during the interviews is a misrepresentation of their actual delivery of the message in practice based on Australian MLHIV KIs’ experiences of suboptimal U = U communication, because Australian MLHIVs were likely referring to experiences with providers other than those in our Australian provider sample. (Of note, we also cannot infer that provider KIs’ delivery of the U = U message when prompted during the interviews *is* an accurate representation of their actual delivery in practice, an additional study limitation.)

Our recruitment sources for the MLHIV and provider KIs who participated in our study were similar but not identical in the two countries. In both countries, the community-based organization that we partnered with to recruit our MLHIV KIs is dedicated to advocating for the rights and wellbeing of PLHIV. However, relative to the US-based organization (Prevention Access Campaign), which focuses primarily on U = U messaging and does so on a global scale, the Australian organization (National Association of People with HIV Australia) emphasizes a broader array of health and advocacy interests of PLHIV and does so at the national level. In both countries, the professional organization/program that we partnered with to recruit our provider KIs supports education and training of health professionals and capacity building in the realm of HIV prevention and care. However, relative to the US-based program (AETC National Coordinating Resource Center), which focuses primarily on HIV, the Australasian organization (ASHM) has a broader focus on other bloodborne viruses and sexual/reproductive health. It is also noteworthy that ASHM is the organization responsible for developing the Australian national clinical guidelines related to U = U [30], which could have contributed to the clearer explanations of U = U among Australian compared with US provider KIs in our sample.

Finally, only six of our 20 provider KIs, all from the US, could be asked about messaging related to “almost zero” sexual transmission risk with low-level viremia given that most interviews had been completed before the literature review and WHO policy brief were published. This recently emergent health communications issue warrants further investigation.

## Conclusions

Collectively, our findings illuminate multiple intervention needs related to U = U messaging among providers, particularly those in the US. First, the message needs to be routinely delivered. Multiple MLHIV KIs from both countries reported that none of their providers had ever spoken to them about U = U or HIV transmission risk despite medical guidelines and ethics codes recommending otherwise [29, 30, 42, 43]. Second, providers should deliver the U = U message using clear and direct language, and they would benefit from the input and feedback of PLHIV and other message recipients regarding word choice and delivery style. They should not assume that patients have pre-existing knowledge of U = U or HIV-related concepts [53]. However, they should also be prepared to tailor the conversation for patients who have advanced knowledge. Visual aids and other resources would be a welcome supplement to provider conversations and may be particularly effective if developed collaboratively with PLHIV and other healthcare users [51, 52, 54]. Third, providers should be aware of the inadvertent harms that conversations about U = U can elicit (e.g., unfounded transmission anxiety, stigma related to viral load detectability) and proactively take measures to circumvent them. For example, they could explain the threshold for transmissibility prior to sharing test results and discuss (un)detectability using non-stigmatizing terms.

Medical education and mandatory or incentivized training can help to ensure that providers’ intervention needs related to communicating with their patients about U = U and evolving HIV science are met. Such initiatives should concomitantly consider and address upstream communication barriers such as disbelief or ambivalence; concerns about patient misunderstanding, non-adherence, and behavior change; vulnerability of clinical judgment to bias and assumptions about patients; apprehension about blame and liability; and uncertainty about the concept of “zero risk.” Emphasizing the value that provider communication about U = U and other HIV risk-related topics adds to the lives of patients living with or without HIV, including numerous psychosocial benefits, may help to further motivate such conversations. Tools such as electronic medical record reminders, checklists, or scripts may be useful, but such tools should be implemented with caution to avoid detracting from the perceived authenticity of the conversation or displacing two-way dialogue [55]. Collaboration with PLHIV and other healthcare users in the process of developing and refining provider communication strategies, tools, and trainings could help to ensure that such outputs are culturally congruent and optimally responsive to patients’ needs in the local community [52, 54].

Ultimately, the success of the U = U messaging campaign can be enhanced or undermined by healthcare providers. Findings of this study can help educators and advocates address providers’ training needs so that providers, in turn, can help their patients realize the benefits of U = U.

## Data Availability

The Framework Method matrix containing all relevant transcript excerpts is available from the lead author (SKC) upon request.

## Code Availability

The analytic framework containing categories, codes, and descriptions is available from the lead author (SKC) upon request.
